# Carbon Black/PDMS Based Flexible Capacitive Tactile Sensor for Multi-Directional Force Sensing

**DOI:** 10.3390/s22020628

**Published:** 2022-01-14

**Authors:** Yinlong Zhu, Xin Chen, Kaimei Chu, Xu Wang, Zhiqiang Hu, Haijun Su

**Affiliations:** 1 College of Mechanical and Electronic Engineering, Nanjing Forestry University, Nanjing 210037, China; ylzhu@njfu.edu.cn (Y.Z.); cx@njfu.edu.cn (X.C.); ckm@njfu.edu.cn (K.C.); 2 State Key Laboratory of Robotics, Shenyang Institute of Automation, Chinese Academy of Sciences, Shenyang 110169, China; hzq@sia.cn; 3 Department of Mechanical and Aerospace Engineering, Ohio State University, Columbus, OH 43210, USA; su.298@osu.edu

**Keywords:** capacitive, tactile sensor, flexible sensor, multi-directional force sensing

## Abstract

Flexible sensing tends to be widely exploited in the process of human–computer interactions of intelligent robots for its contact compliance and environmental adaptability. A novel flexible capacitive tactile sensor was proposed for multi-directional force sensing, which is based on carbon black/polydimethylsiloxane (PDMS) composite dielectric layer and upper and lower electrodes of carbon nanotubes/polydimethylsiloxane (CNTs/PDMS) composite layer. By changing the ratio of carbon black, the resolution of carbon black/PDMS composite layer increases at 4 wt%, and then decreases, which was explained according to the percolation theory of the conductive particles in the polymer matrix. Mathematical model of force and capacitance variance was established, which can be used to predict the value of the applied force. Then, the prototype with carbon black/PDMS composite dielectric layer was fabricated and characterized. SEM observation was conducted and a ratio was introduced in the composites material design. It was concluded that the resolution of carbon sensor can reach 0.1 N within 50 N in normal direction and 0.2 N in 0–10 N in tangential direction with good stability. Finally, the multi-directional force results were obtained. Compared with the individual directional force results, the output capacitance value of multi-directional force was lower, which indicated the amplitude decrease in capacity change in the normal and tangential direction. This might be caused by the deformation distribution in the normal and tangential direction under multi-directional force.

## 1. Introduction

Multi-directional force sensors, especially the flexible ones, play an important role in wearable devices [1,2,3,4,5,6], human-related medical sensing [7,8,9,10], and human–machine interfaces [11,12]. In general, the existing tactile sensors can be classified according to the sensing mechanism employed, including piezo technology [13,14,15,16,17], capacitive technology [18,19], optical technology [20], organic sensing technology [21,22], etc. The principle of capacitive tactile sensor is to convert the measured force information into the variation of capacitance, so as to measure the force by detecting the change of capacitance. Capacitive sensors have high sensitivity and spatial resolution, large pressure range, good dynamic response and simple structure [23,24,25]. However, with the increasing demand for accurate quantitative monitoring, sensors with low sensitivity do not meet the requirements due to the low dielectric constant of dielectric materials. For multi-directional force measurement, sensitivity and measurement range are important factors that should be taken into consideration [26,27,28,29]. Research on the details of the flexible 3D capacitive sensor has been attracting a lot of attention due to its various potential applications [30].

There are many studies on three-axis capacitive tactile sensors [31]. Ting and others [32] proposed a three-directional force tactile sensor based on composite materials. The range and sensitivity of the sensor are good, but the composite material used is expensive and is not suitable for large-scale production and application. Brookhuis et al. [33] fabricated a capacitive three-directional force sensor by micromachining silicon material, which can measure fingertip interaction force, but the system resolution needs to be further improved. Sensors made of flexible material with the advantage of low cost are expected to be investigated for large-scale production and application. Typically, capacitive flexible 3D-force tactile sensors have four elements to obtain the normal and tangential force information [32]. Although capacitive flexible 3D-force tactile sensors are promising candidates for intelligent sensing, there remain difficulties to finding an efficient fabrication procedure to achieve a good balance on simple, low-cost, and precise sensing performances. One convenient solution-based fabrication method with low cost would be more competitive among the solutions. There have been numerous reports on the elastomer-based pressure sensors which include the elastomer composite with other materials such as conductive and inorganic particles [34,35]. The PDMS/carbon material composite is also reported for electrode and dielectric [36,37,38]. Carbon-based conductive materials (such as carbon black and carbon nanotubes) are filled into a polymer matrix (such as polydimethylsiloxane and silica gel) to make composite materials, which are widely used in the research of flexible sensors [39,40,41]. Carbon black is a kind of granular amorphous carbon with a large specific surface area and low price [36]. Compared with carbon black, CNTs are easy to form conductive networks due to their large aspect ratio, which can improve the bonding firmness and reduce the amount of conductive agent [37]. 

In this perspective, we propose a skin-like electronic device through the application of carbon black/PDMS composite dielectric layers. Then, the relationship between the capacitance and force is analyzed theoretically based on a model of a sandwich-type structure under normal/tangential pressure. The sensor is fabricated with carbon black/PDMS composite dielectric layers. In this work, the capacitance sensor is experimentally investigated. The fabrication procedure is low cost due to the solution-based method and the adoption of carbon black as a substitute to relatively expensive CNTs [42,43,44]. The result shows that the sensor has a relatively good sensitivity and spatial resolution, and a proper pressure range with a simple structure, which means it has the potential to be widely equipped in the sensing field of the general robot market.

## 2. Working Principle of Capacitive Tactile Sensors

**Figure 1:**
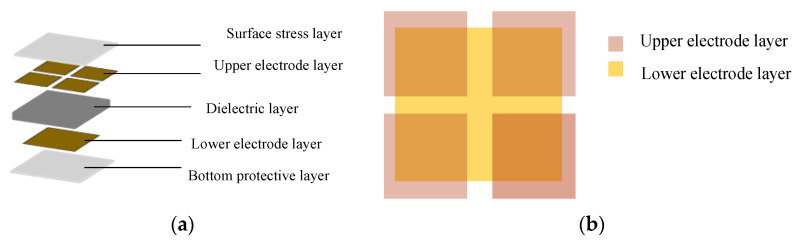
(**a**) Structure scheme of the proposed capacitive sensor; (**b**) projection relationship of upper and lower electrode layers.

The capacitive sensor designed in this paper is mainly composed of a dielectric layer, electrode layer, surface stress layer, and bottom protective layer, as shown in Figure 1a. The dielectric layer is located between the upper and lower plates, and the size of the dielectric layer is 22 mm × 22 mm × 2 mm. The upper electrode consists of four square electrodes of the same size. The size of each square is 10 mm × 10 mm, and the size of the lower electrode is 20 mm × 20 mm as shown in Figure 1b.

In this work, a capacitive prototype including upper and lower electrodes and dielectric layer is manufactured to realize the multi-directional force-sensing function. The principle of parallel plate capacitance is illustrated in Equation (1):

(1) $$C=\frac{{\varepsilon \varepsilon }_{0}S}{d}$$

where ε is the relative permittivity of dielectric, $\varepsilon_{0}$ is the dielectric constant of the vacuum (8.854 × $10^{-12}$ F/m), *S* is the overlapping area of two plates, and *d* is the distance between the two plates.

### 2.1. Working Principle of Normal Force

**Figure 2:**
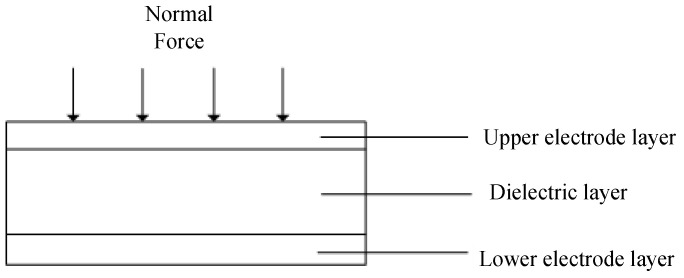
Normal force action testing diagram.

When the sensor is subjected to normal force, the change of capacitance is caused by the change of the distance between the two plates (Figure 2).

It is supposed that the normal force is $F_{Z}$, the initial capacitance of the sensor is $C_{0}$, the initial overlapping area is $S_{0}$, the initial spacing is $d_{0}$, the capacitance after change is *C*, and the distance after change is *d*; then, the variation of the distance is Δd and thus the variation of the capacitance is:

(2) $$\Delta C_{Z}=C-C_{0}=\frac{{\varepsilon \varepsilon }_{0}S_{0}}{d}-\frac{{\varepsilon \varepsilon }_{0}S_{0}}{d_{0}}=\frac{{\varepsilon \varepsilon }_{0}S_{0}\Delta d}{{\mathrm{dd}}_{0}}$$

The variation of spacing Δd can be expressed as follows:

(3) $$\Delta {d=d}_{0}-\;d$$

The relationship between capacitance variation and distance variation is as follows:

(4) $$\frac{\Delta C_{Z}}{C_{0}}=\frac{\Delta d}{d_{0}-\Delta d}$$

Common constitutive models include the Yeoh model, Mooney–Rivlin model, and Ogden model. The Yeoh model is more suitable for simulating the large deformation behavior of materials and the material parameters can be obtained through tensile tests. The Yeoh model is selected as the constitutive model of PDMS. It is assumed that the PDMS material is homogeneous and incompressible which exhibits nonlinear hyperelastic behavior. The constitutive model of PDMS materials is described by the strain energy density function w = w($I_{1}$,${\;I}_{2}$,${\;I}_{3}$), where $I_{1}$ is the first invariant of the stress tensor, $I_{2}$ is the second invariant of the stress tensor, and $I_{3}$ is the third invariant of the stress tensor, which can be defined as:

(5) $$\{\begin{array}{c} I_{1}=\lambda_{1}^{2}+\lambda_{2}^{2}+\lambda_{3}^{2}\\ I_{2}=\lambda_{1}^{2}\lambda_{2}^{2}+\lambda_{2}^{2}\lambda_{3}^{2}+\lambda_{1}^{2}\lambda_{3}^{2}\\ I_{3}=\lambda_{1}^{2}\lambda_{2}^{2}\lambda_{3}^{2} \end{array}$$

$\lambda_{1},{\;\lambda }_{2},{\;\lambda }_{3}$ are the main elongation ratios. PDMS materials are not compressible, $I_{3}$ = 1. When the sensor is under pressure on the normal force of the *x*, *y*, *z* directional, respectively:

(6) $$\lambda_{1}=\lambda_{2}$$

$\lambda_{3}$ can be expressed as:

(7) $$\lambda_{3}\;=\frac{1}{\lambda_{1}^{2}}$$

The main extension ratio can be expressed as:

(8) $$\lambda_{3}\;=\frac{d}{d_{0}}$$

The strain energy density function of the Yeoh model can be expressed as:

(9) $$W=\sum_{i=1}^{3}C_{i0}{\left(I_{1}-3\right)}^{i}+\sum_{i=1}^{3}\frac{1}{D_{i}}{\left(J-1\right)}^{2i}$$

where $C_{i0}$ and $D_{i}$ are undetermined coefficients, and J is the elastic volume ratio. It can be obtained through the tensile test of the material *C*_10_ = 0.03 and *C*_20_ = 0.3. PDMS materials are not compressible, thus J=1. Some papers suggest that the 2nd or 3rd order Yeoh model is better for compression [45,46]. In this work, the 2nd order Yeoh model is used. The typical binomial parameter form of the Yeoh model is as follows:

(10) $$W=C_{10}\left(I_{1}-3\right)+C_{20}{\left(I_{1}-3\right)}^{2}$$

The true principal stress $\sigma_{i}\left(i=1,\;2,\;3\right)$ can be obtained by calculating the partial derivative of the strain energy density function with respect to the principal elongation ratio $\lambda_{i}$:

(11) $$\sigma_{i}=\lambda_{i}\frac{\partial W}{\partial \lambda_{i}}-p=2\lambda_{i}^{2}\left[C_{10}+2C_{20}{\left(I_{1}-3\right)}^{2}\right]-p$$

p is the hydrostatic pressure, which can be determined by incompressible conditions. The sensor only deforms in the direction of the normal force, $\sigma_{1}=\sigma_{2}=0$, so the hydrostatic pressure p is:

(12) $$p=2\lambda_{1}^{2}\left[C_{10}+2C_{20}{\left(I_{1}-3\right)}^{2}\right]$$

The principal stress $\sigma_{3}$ in the direction of the normal force is:

(13) $$\sigma_{3}=2(\lambda_{3}^{2}-\lambda_{1}^{2})\left[C_{10}+2C_{20}{\left(I_{1}-3\right)}^{2}\right]$$

Substituting Equations (5) and (7) into Equation (13) can obtain:

(14) $$\sigma_{3}=2(\lambda_{3}^{2}-\frac{1}{\lambda_{3}})\left[C_{10}+2C_{20}{\left(\frac{2}{\lambda_{3}}+\lambda_{3}^{2}-3\right)}^{2}\right]$$

The stress can be expressed as:

(15) $$\sigma =\frac{F}{S}$$

It can be obtained from the law of volume invariance:

(16) $$S_{0}d_{0}=Sd$$

Substituting Equations (8), (15) and (16) into Equation (14) can obtain:

(17) $$F=2\frac{S_{0}d_{0}}{d}\left(\frac{d^{2}}{d_{0}^{2}}-\frac{d_{0}}{d}\right)\left[C_{10}+2C_{20}{\left(\frac{2d_{0}}{d}+\frac{d^{2}}{d_{0}^{2}}-3\right)}^{2}\right]$$

Substituting Equations (3) and (4) into Equation (17) can obtain:

(18) $$F\;=\;2S_{0}\;(\frac{\Delta C_{Z}}{C_{0}}+1)[\frac{1}{{\left(\frac{\Delta C_{Z}}{C_{0}}+1\right)}^{2}}-\left(\frac{\Delta C_{Z}}{C_{0}}+1\right)]\{C_{10}+2C_{20}{\left[2\left(\frac{\Delta C_{Z}}{C_{0}}+1\right)+\frac{1}{{\left(\frac{\Delta C_{Z}}{C_{0}}+1\right)}^{2}}-3\right]}^{2}\}$$

According to Equation (18), the relationship between the normal force $F_{Z}$ and the relative capacitance variation $\frac{\Delta C_{Z}}{C_{0}}$ can be obtained when the initial capacitance $C_{0}$ the initial overlapping area $S_{0}$ and $C_{10}$,${\;C}_{20}$ are known.

The capacitance change in *Z* direction $\Delta C_{Z}$ can be represented by $C_{11},{\;C}_{12},{\;C}_{21},{\;C}_{22}$:

(19) $$\Delta C_{Z}=\frac{\Delta C_{11}+\Delta C_{12}+\Delta C_{21}+\Delta C_{22}}{4}$$

### 2.2. Working Principle of Tangential Force

**Figure 3:**
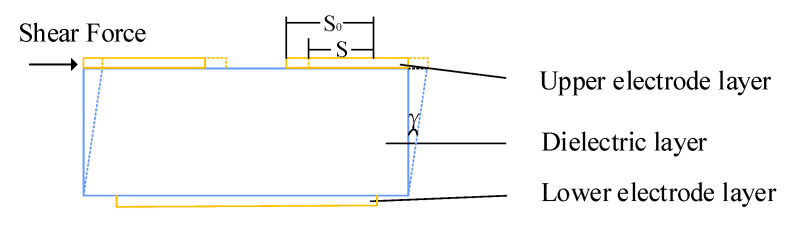
Tangential force testing diagram.

When the sensor is subjected to tangential force, the change of capacitance is caused by the change of the overlapping area between both plates (Figure 3).

It is assumed that the tangential force is $F_{X}$, the initial capacitance of the sensor is $C_{0}$, the initial overlapping area is $S_{0}$, the initial spacing is $d_{0}$, the capacitance after change is *C*, the overlapping area after change is *S*, and the overlapping area change is Δ*S*; thus, the capacitance change $\Delta C_{X}$ is:

(20) $$\Delta C_{X}=C-C_{0}=\frac{{\varepsilon \varepsilon }_{0}S_{0}}{d}-\frac{{\varepsilon \varepsilon }_{0}S_{0}}{d_{0}}=\frac{{\varepsilon \varepsilon }_{0}S_{0}\Delta d}{{\mathrm{dd}}_{0}}$$

The overlapping area change ΔS can be expressed as follows:

(21) $$\Delta {S=S}_{0}-S$$

The relationship between the capacitance change of the sensor and the overlapping area change is as follows:

(22) $$\frac{\Delta C_{X}}{C_{0}}=\frac{\Delta S}{S_{0}}$$

According to the shear Hooke’s law of material mechanics, the relationship between shear stress and shear strain is as follows:

(23) τ=Gγ

G is the proportional constant, called the shear modulus of the material, which can be expressed by the elastic modulus *E* and Poisson’s ratio

(24) $$G\;=\frac{E}{2\left(1+\mu \right)}$$

The shear stress can be expressed as:

(25) $$\tau =\frac{F_{X}}{S_{0}}$$

Because the shear strain γ is very small, it can be expressed as:

(26) γ ≈ tan γ

When Equations (23)–(25) are taken into Equation (21), the following results can be obtained:

(27) $$\Delta S=\frac{{2d}_{0}\left(1+\mu \right)}{E\sqrt{S_{0}}}F_{X}-{\left[\frac{{2d}_{0}\left(1+\mu \right)}{S_{0}E}\right]}^{2}F_{X}{}^{2}$$

By introducing Equation (27) into Equation (22), the following equation can be obtained:

(28) $$\frac{\Delta C_{X}}{C_{0}}=\frac{{2d}_{0}\left(1+\mu \right)}{{\mathrm{ES}}_{0}{}^{\frac{3}{2}}}[F_{X}-\frac{2d_{0}\left(1+\mu \right)}{{\mathrm{ES}}_{0}{}^{\frac{3}{2}}}{\;F}_{X}{}^{2}]$$

According to Equation (28), when the initial capacitance $C_{0}$, initial spacing $d_{0}$, elastic modulus *E* of dielectric layer and Poisson’s ratio μ are given, the relationship between the magnitude of tangential force $F_{X}$ and capacitance variation $\Delta C_{X}$ can be obtained (Equation (29)). With the same method, the tangential force in *Y* direction $F_{Y}$ can be obtained.

(29) $$F_{X}=\frac{{\mathrm{ES}}_{0}{}^{\frac{3}{2}}(1-\sqrt{1-4\;\frac{\Delta C_{X}}{C_{0}}})}{{4d}_{0}\left(1+\mu \right)}$$

The capacitance change in *X* direction $\Delta C_{X}$ can be represented by $\Delta C_{11},\;\Delta C_{12},\;\Delta C_{21},$ $\Delta C_{22}$:

(30) $$\Delta C_{X\;}=\frac{\left(\Delta C_{12}+\Delta C_{22}\right)-\;(\Delta C_{11}+\Delta C_{21})}{4}$$

By introducing Equation (30) into Equation (29), we can get the following result:

(31) $$F_{X}=\frac{{\mathrm{ES}}_{0}{}^{\frac{3}{2}}(1-\sqrt{1-\frac{\left(\Delta C_{12}+\Delta C_{22}\right)-(\Delta C_{11}+\Delta C_{21})}{C_{0}}})}{{4d}_{0}\left(1+\mu \right)}$$

The capacitance change in *Y* direction $\Delta C_{Y}$ can be represented by $\Delta C_{11},\;\Delta C_{12}\;,\;\Delta C_{21\;},\;\Delta C_{22}$:

(32) $$\Delta C_{Y}=\frac{\left(\Delta C_{21}+\Delta C_{22}\right)\;\;-\;(\Delta C_{11}+\Delta C_{12})}{4}$$

By introducing Equation (32) into Equation (29), we can get the following result:

(33) $$F_{Y}=\frac{{\mathrm{ES}}_{0}{}^{\frac{3}{2}}\left(1\;-\;\sqrt{1\;-\;\frac{\left(\Delta C_{21}+\Delta C_{22}\right)\;-\;(\Delta C_{11}+\Delta C_{12})}{C_{0}}}\right)}{{4d}_{0}\left(1+\mu \right)}$$

## 3. Fabrication Process of the Soft Capacitive Sensor

### 3.1. Preparation of Electrode Layer

**Figure 4:**
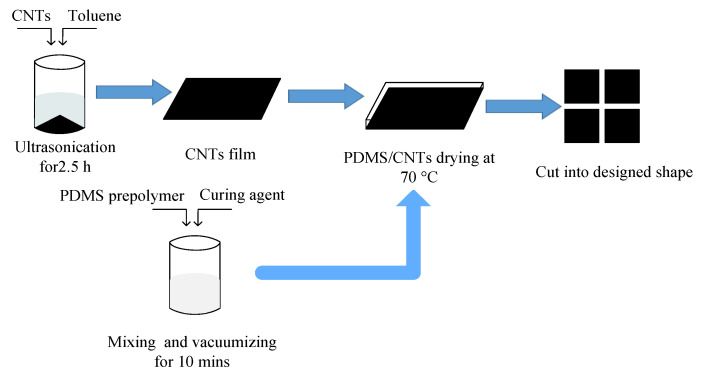
Schematic diagram of electrode layer preparation.

Carbon nanotubes (CNTs) were selected as electrode layer conductive materials. The electrode layer preparation process was (Figure 4): first (i) PDMS prepolymer was mixed with a curing agent at the mass ratio of 10:1 and then it was stirred with a glass rod and put into a magnetic mixer for stirring and vacuumizing for 10 min to remove bubbles; (ii) 0.1 g of CNTs were dispersed in 10 g of toluene solvent and then ultrasonically dispersed in an ultrasonic cleaning machine (power: 240 W) for 2.5 h to obtain the carbon nanotube dispersion, and the carbon nanotube dispersion was evenly dripped on the coater at 70 °C until uniform carbon nanotube film was formed; and (iii) the prepared PDMS solution was evenly covered on the surface of the carbon nanotube film and cured at 70 °C, and then the CNTs/PDMS film was peeled from the coating machine and cut into the required shape and size as the electrode layer.

### 3.2. Preparation of Dielectric Layer

**Figure 5:**
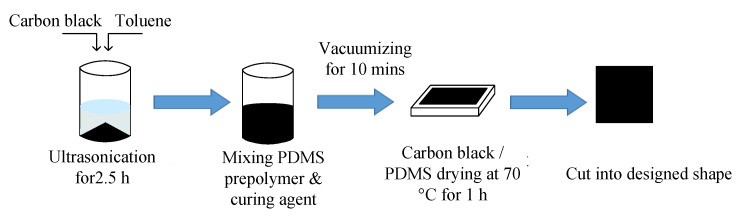
Schematic diagram of dielectric layer preparation.

Polydimethylsiloxane (PDMS) with micro carbon black particles inside was selected as the dielectric layer of the soft capacitive sensor. The preparation process of the dielectric layer was (Figure 5): first (i) carbon black with different mass fractions was dispersed in toluene solvent and ultrasonically dispersed for 2.5 h in an ultrasonic cleaning machine to obtain carbon black dispersion solution; then, (ii) PDMS prepolymer was added. The beaker was placed on an electric heating plate and heated at 60 °C until toluene volatilized completely. Whether toluene had been completely volatilized can be judged by the weighing method. The mass ratio of prepolymer to curing agent is 10:1. The curing agent is added in the proportion of 1:1 and is stirred with a glass rod and then put into a magnetic stirrer for 10 min to remove bubbles; then, (iii) it is poured into the mold and put into the blast drying oven. The temperature is set at 70 °C for 1 h of drying. Finally, the carbon black/PDMS composite is cut into the required shape and size as the medium layer. Multi-directional force detection can also be realized by thermal drawing—a very simple method [47,48]. 

### 3.3. Morphology Characterization

**Figure 6:**
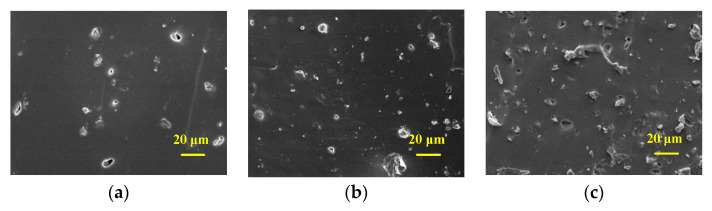
SEM observation of carbon black/PDMS composite: (**a**) carbon black content of 0.5 wt%; (**b**) carbon black content of 2 wt%; and (**c**) carbon black content of 4 wt%.

Some carbon black/PDMS composites with partial mass fraction were selected to observe their morphology characterization. The cross-section of the composite dielectric material was cut off. After gold spraying under vacuum, the morphology of carbon black in carbon black/PDMS composite cross-section in PDMS was observed by scanning electron microscope at 10 kV. The cold field electron microscope pictures were illustrated (Figure 6) and the carbon black content in Figure 6a is 0.5 wt%. The carbon black content of Figure 6b is 2 wt%, and that of Figure 6c is 4 wt%. Carbon black particles are bright dots shown in the images, and PDMS is represented as the dark area.

It can be seen that there are obvious differences between carbon black/PDMS composites with different carbon black content. With the increase in carbon black mass fraction, the mixed carbon black on the surface of carbon black/PDMS composites also increases. Figure 6a shows the characterization of carbon black/PDMS composite mixed with 0.5 wt%, and it can be seen that there are only a few carbon black particles on the surface of the film. Figure 6b shows that the carbon black/PDMS composite with 2 wt% is evenly distributed. In Figure 6c, there is a significant difference when the carbon black reached 4 wt%, for certain connectivity of carbon black in PDMS has occurred.

### 3.4. Dielectric Constant Test

**Figure 7:**
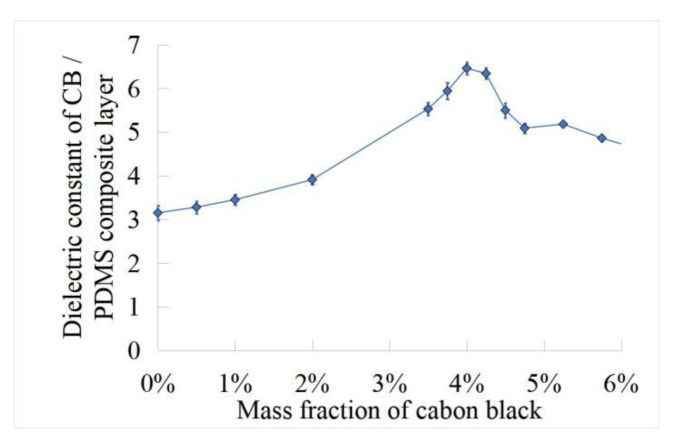
Dielectric constant of carbon black/PDMS composites with different mass fractions.

In order to obtain carbon black/PDMS composite dielectric layer with excellent dielectric properties, the capacitance of carbon black/PDMS composite dielectric layer with different mass fractions (0.5 wt%, 2 wt%, 3 wt%, 3.5 wt%, 3.75 wt%, 4 wt%, 4.25 wt%, 4.5 wt%, 5 wt%, and 6 wt%) was measured by LCR bridge. As shown in Figure 7, the test frequency is 100 kHz and the test voltage is 1 V. It can be concluded that the dielectric constant of carbon black/PDMS composite layer increases at 4 wt% and then decreases. The results show that the dielectric properties of carbon black/PDMS composite layer are the best at 4 wt%. In Figure 7, we demonstrate the dielectric constant of carbon black/PDMS composite layers according to the weight concentration of carbon black. The addition amount of the conductive agent follows the percolation theory of the conductive particles in the polymer matrix; that is, the conductive agent reaches the optimal polarization characteristic state in the polymer matrix after addition up to a certain amount, and the dielectric property of the composite material cannot be significantly improved if it continues to be added.

For carbon black/PDMS composites, when the mass fraction of carbon black in PDMS reaches a critical value, the carbon black/PDMS composite will change from unconnected to connective gradually, which puts the carbon black/PDMS composite in a transition state between insulator and conductor, and the conductivity of the carbon black/PDMS composite may change suddenly. Thus, the dielectric properties of the composite materials are improved, which can be used as the dielectric layer of the sensor to optimize the performance of the sensor. When the sensor is under pressure, the distance between the upper and lower electrodes changes, and the carbon black particles form a certain connection in PDMS, which makes the conductivity and effective dielectric coefficient of the composite increase. Therefore, filling carbon black particles in PDMS can increase the effective dielectric coefficient of the dielectric layer, thus improving the sensitivity of the sensor. However, although carbon black particles increase the dielectric coefficient of carbon black/PDMS composite dielectric layer, it also increases the Young’s modulus of the composite material. Young’s modulus will affect the overall flexibility of the composite dielectric layer, resulting in the reduction in dielectric layer deformation under pressure, which affects the sensitivity of the sensor. With the increase in viscosity, the sensor recovers slowly after receiving the pressure, which increases the hysteresis of the sensor. Therefore, in the preparation of carbon black/PDMS composites, it is very important to reasonably select the carbon black content for the performance of the sensor.

### 3.5. Preparation of Surface Stress Layer

PDMS was mixed with prepolymer and curing agent at the ratio of 8:1 by mass. The mixture was first stirred with a glass rod and then put into a magnetic mixer for stirring and vacuumizing for 10 min to remove bubbles. The PDMS film was poured into the mold and put into the blast drying oven. The temperature was set at 70 °C for 1 h until drying. Finally, the PDMS film was cut into the required shape and size as the surface stress layer.

### 3.6. Assembly of Sensor

**Figure 8:**
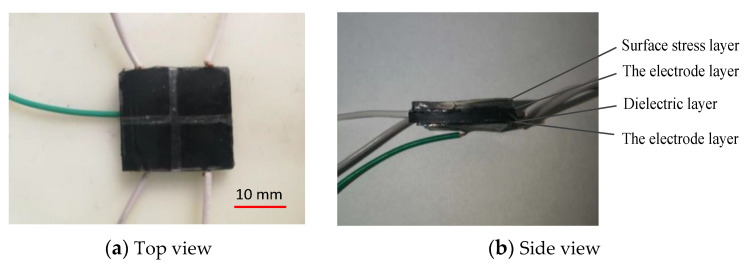
Physical picture of sensor.

The electrode layer, dielectric layer, and surface stress layer made and cut are placed in alignment, and fixed together by the mixed prepolymer and curing agent of PDMS to obtain the capacitive flexible sensor. The CNT/PDMS film is used as the electrode layer, and carbon black/PDMS composite material is used as the dielectric layer (Figure 8).

## 4. Experimental Setup

### 4.1. Measurement Experiment of Sensor Normal Force

**Figure 9:**
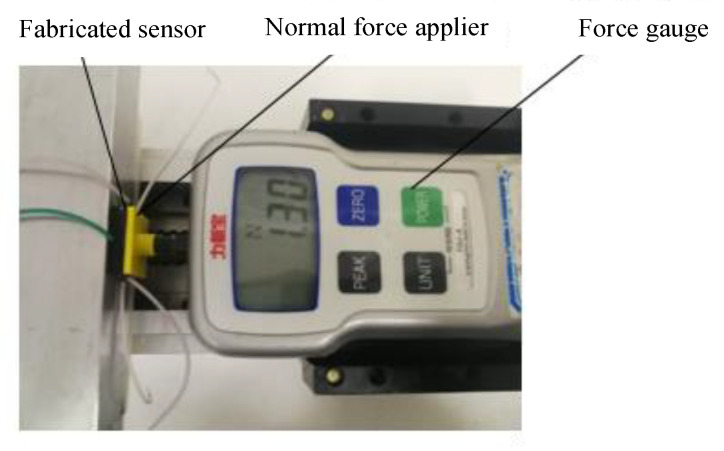
Normal force test platform.

A normal force was applied on the sensor with a test platform (Figure 9). A force gauge was used to apply pressure to the sensor in the normal direction, and the output capacitance values under different normal forces are recorded. In the experiment, the force is applied at the speed of 1 mm/s.

**Figure 10:**
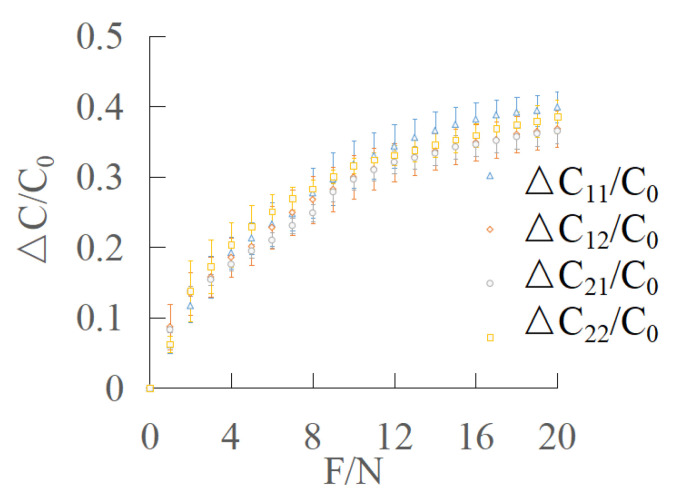
Output characteristic curve of normal force.

The effective range of normal force is 0–20 N. The normal force is loaded every 1 N, and the average is obtained by repeated loading for 5 times. The output characteristic curve of the sensor is smooth (Figure 10). The standard deviation values of the normal force are listed (Table 1).

It can be seen that when pressure is applied to the surface stress layer of the sensor, the capacitance changes of $C_{11}{,\;C}_{12}{,\;C}_{21}{,\;C}_{22}$ show an upward trend with the increase in normal force applied in the Z direction. This is because the surface stress layer produces the same pressure distribution and the corresponding deformation of the dielectric layer. Due to the symmetry of the structure, the compression deformation of the dielectric layer corresponding to the four capacitors is the same; that is, the spacing *d* of the four capacitors decreases at the same time, which makes the four capacitance values increase and change the same.

**Figure 11:**
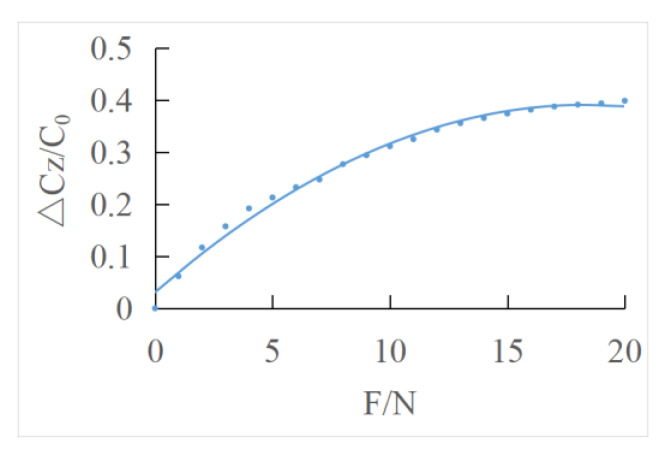
Least squares fitting the output characteristic curve of normal force direction.

The output characteristic curve of normal force direction is polynomial fitted by the least square method (Figure 11). $\Delta C_{Z}$ can be represented by $C_{11}{,\;C}_{12}{,\;C}_{21}{,\;C}_{22}$ according to Equation (19).

The fitting curve equation of the least square method is as follows:

(34) $$(\frac{\Delta C}{C_{0}})=-0.0009\;F^{2}+0.0343\;F+0.0032$$

**Figure 12:**
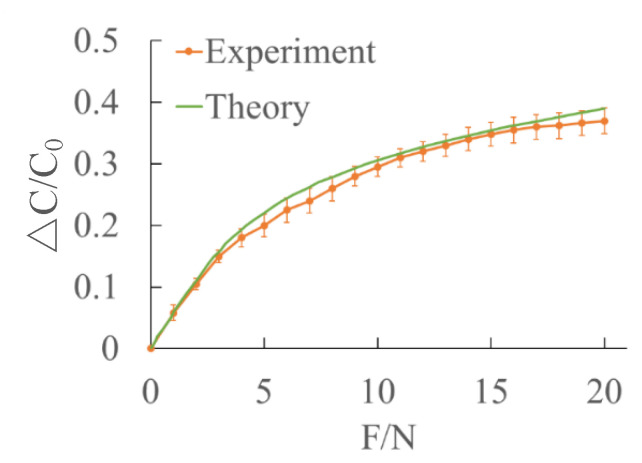
Comparison of experimental data and theoretical data under normal force loading.

The experimental curves are compared with the theoretical values (Figure 12). It can be seen that the theoretical results agree well with the experimental one.

### 4.2. Experiment on Measuring Tangential Force of Sensor

The tangential force is divided into *X* and *Y* directions. A push–pull gauge is used to apply pressure to the sensor in *X* and *Y* directions, respectively, and output capacitance value under different tangential force is recorded.

**Figure 13:**
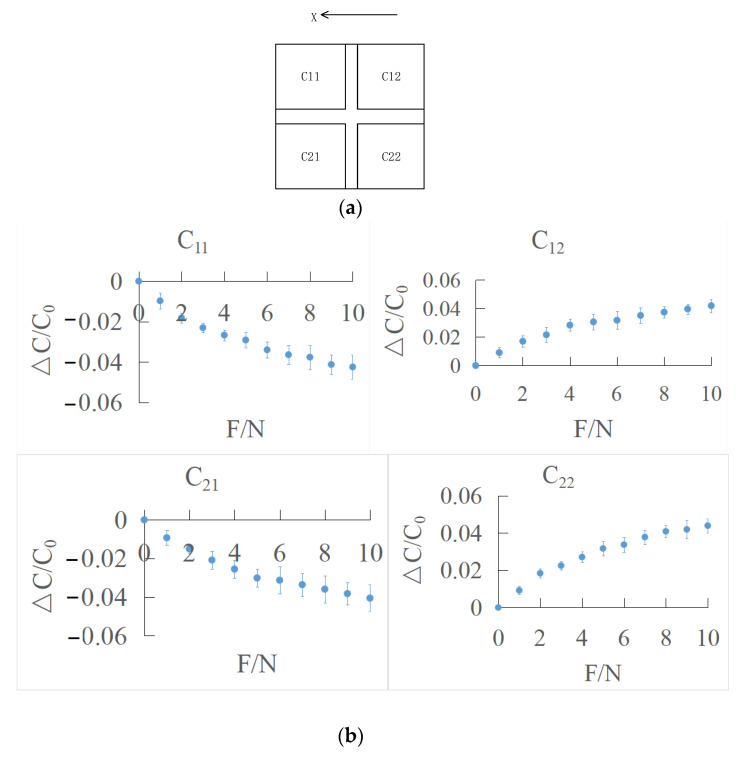
(**a**) Schematic diagram of X direction; (**b**) output characteristic curve of tangential force in *X* direction.

The schematic diagram of the *X* direction is shown in Figure 13a. The effective range of tangential force in the *X* direction is 0–10 N. The tangential force is loaded every 1 N, and the average is obtained by repeated loading for 5 times. The output characteristic curve of the sensor is shown in Figure 13b.

It can be seen that $C_{12}$ and $C_{22}$ show an upward trend, while $C_{11}$ and $C_{21\;}$ present a downward trend. When the tangential force is applied to the stress layer on the sensor surface, the surface stress layer is pushed, which drives the dielectric layer to deform and changes the overlapping area of the upper and lower electrodes. According to the experiment results, the overlapping area, *S*, of two capacitors, $C_{12}$ and $C_{22}$, close to the force application increases at the same time, while the positive opposite area, s, of two capacitors, $C_{11}$ and $C_{21\;}$, far away from the force application point decreases at the same time, which makes the four capacitance values change in the same amount.

**Figure 14:**
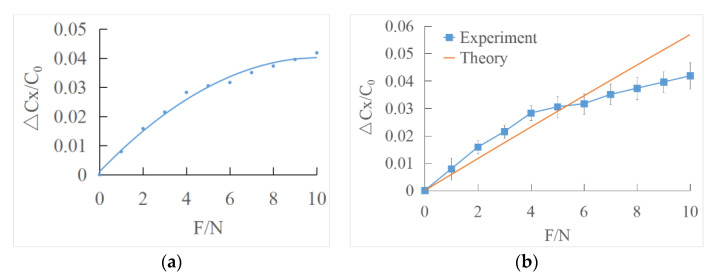
(**a**) Least squares fitting the output characteristic curve of tangential force direction; (**b**) comparison of experimental data and theoretical data under shear force loading.

The least square method is used to polynomial fit the output characteristic curve of tangential force direction (Figure 14a).

The fitting curve equation of the least square method is as follows:

(35) $$(\frac{\Delta C}{C_{0}})=-0.0004\;F^{2}+0.0077\;F+0.0011$$

The experimental curve was compared with the theoretical value (Figure 14b). $\Delta C_{X}$ can be represented by $\Delta C_{11},\;\Delta C_{12},\;\Delta C_{21},\;\Delta C_{22}$ according to Equation (30). When the applied force was over about 5 N, there is a significant difference between the experimental and theoretical value, and the measured tangential force becomes lower. The reason might be that when the applied tangential force reaches a certain value, there is a small slide between the interface. Thus, the actual force becomes smaller, which leads to the decrease in the capacitance variance.

## 5. Results and Discussion

For carbon black/PDMS composites, when the mass fraction of carbon black in PDMS reaches a critical value, the carbon black/PDMS composite will change from unconnected to connective gradually, which puts the carbon black/PDMS composite in a transition state between insulator and conductor, and the conductivity of the carbon black/PDMS composite may change suddenly. Thus, the dielectric properties of the composite materials are improved, which can be used as the dielectric layer of the sensor to optimize the performance of the sensor.

When the sensor is under pressure, the distance between the upper and lower electrodes changes, and the carbon black particles form a certain connection in PDMS, which makes the conductivity and effective dielectric coefficient of the composite increase. Therefore, filling carbon black particles in PDMS can increase the effective dielectric coefficient of the dielectric layer, thus improving the sensitivity of the sensor. However, although carbon black particles increase the dielectric coefficient of the carbon black/PDMS composite dielectric layer, it also increases the Young’s modulus of the composite material. Young’s modulus will affect the overall flexibility of the composite dielectric layer, resulting in the reduction in dielectric layer deformation under pressure, which affects the sensitivity of the sensor. With the increase in viscosity, the sensor recovers slowly after receiving the pressure, which increases the hysteresis of the sensor. Therefore, in the preparation of carbon black/PDMS composites, it is very important to select the carbon black content reasonably for the performance of the sensor.

### 5.1. Sensitivity of Sensor

The minimum detectable force is 0.1 N in normal direction and 0.2 N in tangential direction.

The sensitivity of the sensor can be expressed by the ratio of the relative change of capacitance to the change of force. The sensitivity of the sensor can be expressed as

(36) $$S=\frac{\Delta C}{C_{0}\Delta F}$$

The range of the sensor in Z direction is 0–20 N. The relative change of capacitance between 0 and 9 N is 0.725, so the sensitivity of normal force direction between 0 and 9 N is 0.028 $N^{-1}$. The relative change of capacitance between 10 N and 20 N is 0.179, so the sensitivity of normal force direction between 10 N and 20 N is 0.0049 $N^{-1}$.

The range of the sensor in X and Y direction is 0–10 N. The relative change of capacitance between 0 N and 5 N is 0.0675, so the sensitivity of tangential force direction between 0 N and 5 N is 0.0061 $N^{-1}$. The relative change of capacitance between 6 N and 10 N is 0.021, so the sensitivity of tangential force direction between 6 N and 10 N is 0.0019 $N^{-1}$. Comparison of the proposed sensor with different sensors was illustrated in Table 2.

### 5.2. Fatigue of Sensor

**Figure 15:**
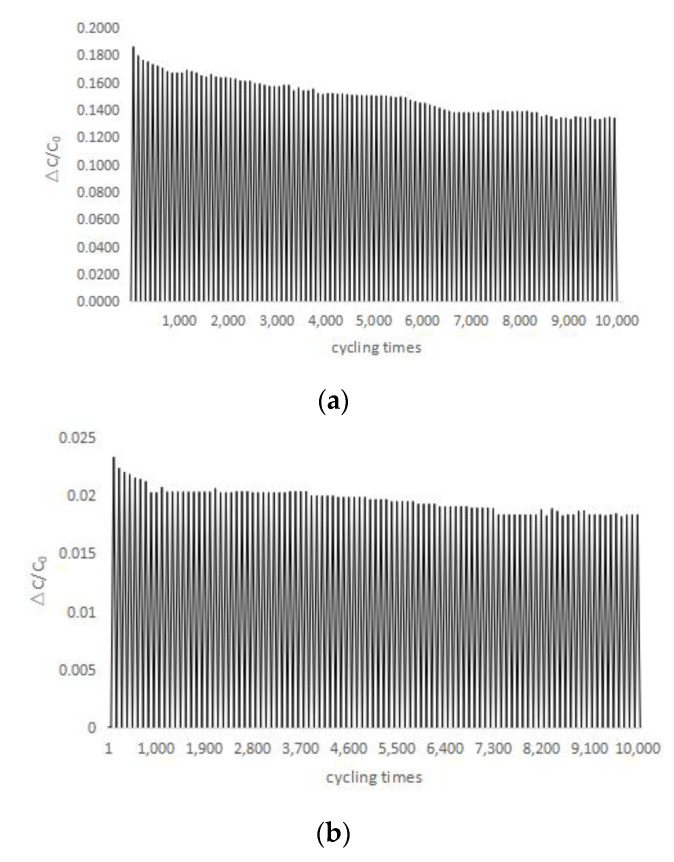
Fatigue of sensor under (**a**) normal pressure and (**b**) tangential pressure.

A force of 3 N is applied to the sensor for 10,000 pressure loading and unloading cycles. LCR bridge is used to measure the relative capacitance changes of normal force and tangential force of the sensor (Figure 15); it can be seen that the relative variation of capacitance of the sensor is basically stable. For the normal force, ΔC/C0 decreased from 0.18 to 0.14 after 10,000 cycles. For the tangential force, the ΔC/C0 decreased from 0.023 to 0.018 after 10,000 cycles. The value of ΔC/C0 decreased rapidly in the first 1000 cycles, then it reached a relative stable value after about 7500 cycles, indicating that the sensor has good stability after about 7500 cycles. According to the fatigue performance, compensation ought to be used to obtain the precise value.

### 5.3. The Test of Multidirectional Force

**Figure 16:**
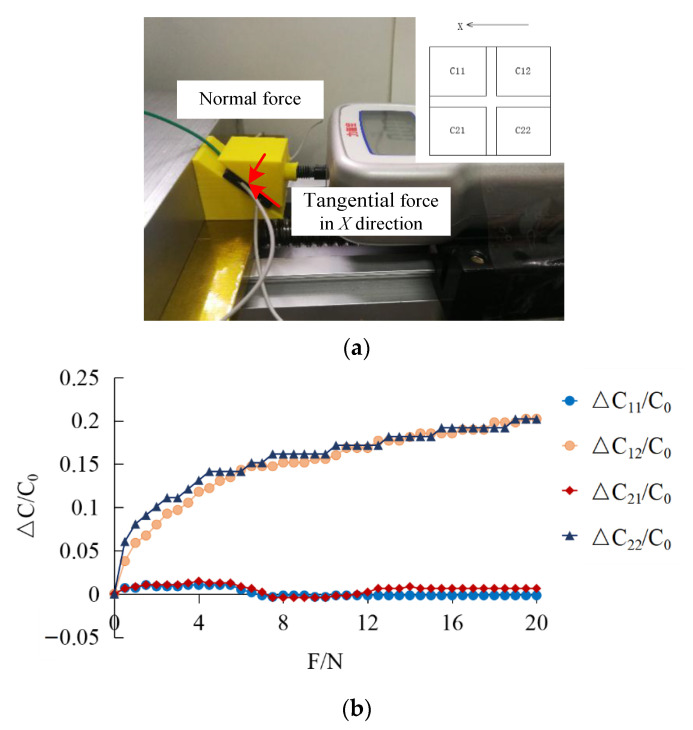
(**a**) Multidirectional force test platform; (**b**) output capacitance value of multi-directional force.

A multi-directional force with an inclination angle of 45° was applied to the sensor surface, as shown in Figure 16a. A multi-directional force of 0–20 N was applied on the sensor surface with a push–pull gauge and the real-time output capacitance values of $C_{11\;}{,\;C}_{12\;}{,\;C}_{21}{\;\mathrm{and}\;C}_{22}$, were recorded as shown in Figure 16b. Due to the coupling of the multi-directional loadings, the deformation would be more complex. Here, according to Equations (19) and (30), the magnitude of the normal force and the shear force could be obtained. The normal force could be reflected by $C_{12}$ and $C_{22}$, the shear force could be reflected by $C_{11}$ and $C_{21}$. It can be seen that the changes of $C_{12}$ and $C_{22}$ are consistent, showing an upward trend, while the changes of $C_{11}$ and $C_{21}$ are the same, increasing first and then decreasing. The relative variation of capacitance of multi-directional force is lower than that of normal force, which may be due to the simultaneous application of normal force and tangential force. The application of normal force hinders the deformation in the direction of tangential force, and the application of tangential force hinders the deformation in the direction of normal force.

## 6. Conclusions

A novel flexible capacitive tactile sensor was proposed for multi-directional force sensing based on a carbon black/PDMS composite dielectric layer and upper and lower electrodes of a CNTs/PDMS composite layer. The theoretical model of the flexible multi-directional force sensor was established, which could predict the normal directional force more precisely than the tangential directional force. By changing the ratio of carbon black, it was found that the dielectric constant of the carbon black/PDMS composite layer increases at 4 wt%, and then decreases. Then, the prototype with a carbon black/PDMS composite dielectric layer was fabricated and characterized. According to the performed experiments, the minimum detectable force is 0.1 N in normal direction and 0.2 N in tangential direction. It has the potential to achieve good performance in a low-cost method with the proposed convenient solution-based fabrication method. In the design of the dielectric layer, carbon black could be chosen as an economical addition material in substitution of CNTs, which could lead to a general adoption of flexible sensing.

## Figures and Tables

**Table 1:** The standard deviation values of the normal force.

FORCE/N	Standard Deviation (*C*_11_)	Standard Deviation (*C*_12_)	Standard Deviation (*C*_21_)	Standard Deviation (*C*_22_)
0	0	0	0	0
1	0.0123	0.0317	0.0344	0.0116
2	0.0233	0.0304	0.0286	0.0435
3	0.0301	0.0286	0.0312	0.0382
4	0.0229	0.0272	0.0327	0.0318
5	0.0226	0.0270	0.0272	0.0310
6	0.0313	0.0301	0.0300	0.0241
7	0.0243	0.0317	0.0275	0.0164
8	0.0356	0.0335	0.0132	0.0128
9	0.0370	0.0312	0.0198	0.0094
10	0.0331	0.0312	0.0230	0.0114
11	0.0327	0.0297	0.0257	0.0105
12	0.0308	0.0271	0.0249	0.0137
13	0.0268	0.0258	0.0252	0.0155
14	0.0268	0.0259	0.0250	0.0174
15	0.0249	0.0255	0.0250	0.0181
16	0.0231	0.0257	0.0243	0.0169
17	0.0211	0.0258	0.0231	0.0163
18	0.0211	0.0259	0.0240	0.0181
19	0.0216	0.0260	0.0254	0.0223
20	0.0216	0.0260	0.0255	0.0237

**Table 2:** Comparison of the proposed sensor with different sensors.

References	Electrode Material	Dielectric Layer	Sensitivity
Fu [49]	Gold/PI	CNT/PDMS	0.0161/kPa
Kai Ke [50]	Ag	CNS/GNP/TPU	0.00205/kPa
This work	CNTs/PDMS	CB/PDMS	0.0196/kPa

## Data Availability

All test data mentioned in this paper will be made available on request to the correspondent author’s email with appropriate justification.
